# Coronary artery bypass surgery compared with percutaneous coronary interventions in patients with insulin-treated type 2 diabetes mellitus: a systematic review and meta-analysis of 6 randomized controlled trials

**DOI:** 10.1186/s12933-015-0323-z

**Published:** 2016-01-06

**Authors:** Pravesh Kumar Bundhun, Zi Jia Wu, Meng-Hua Chen

**Affiliations:** Institute of Cardiovascular Diseases, the First Affiliated Hospital of Guangxi Medical University, Nanning, Guangxi 530027 Peoples’ Republic of China

**Keywords:** Percutaneous coronary intervention, Coronary artery bypass surgery, Insulin-treated diabetes mellitus, Adverse clinical outcomes

## Abstract

**Background:**

Data regarding the long-term clinical outcomes in patients with insulin-treated type 2 diabetes mellitus (ITDM) revascularized by either coronary artery bypass surgery (CABG) or percutaneous coronary intervention (PCI) are still controversial. We sought to compare the long-term (≥1 year) adverse clinical outcomes in patients with ITDM who underwent revascularization by either CABG or PCI.

**Methods:**

Randomized Controlled Trials (RCTs) comparing the long-term clinical outcomes in patients with ITDM and non-ITDM revascularized by either CABG or PCI were searched from electronic databases. Data for patients with ITDM were carefully retrieved. Odd Ratio (OR) with 95 % confidence interval (CI) was used to express the pooled effect on discontinuous variables and the pooled analyses were performed with RevMan 5.3.

**Results:**

Six RCTs involving 10 studies, with a total of 1297 patients with ITDM were analyzed (639 patients from the CABG group and 658 patients from the PCI group). CABG was associated with a significantly lower mortality rate compared to PCI with OR: 0.59, 95 % CI 0.42–0.85; P = 0.004. Major adverse cardiovascular and cerebrovascular events as well as repeated revascularization were also significantly lower in the CABG group with OR: 0.51, 95 % CI 0.27–0.99; P = 0.03 and OR 0.34, 95 % CI 0.24–0.49; P < 0.00001 respectively. However, compared to PCI, the rate of stroke was higher in the CABG group with OR: 1.41, 95 % CI 0.64–3.09; P = 0.40, but this result was not statistically significant.

**Conclusion:**

CABG was associated with significantly lower long-term adverse clinical outcomes compared to PCI in patients with ITDM. However, due to an insignificantly higher rate of stroke in the CABG group, further researches with a larger number of randomized patients are required to completely solve this issue.

## Background

Type 2 Diabetes mellitus (T2DM) and Coronary Artery Disease (CAD) are two important and prevalent chronic disorders which often co-exist [1]. Patients with T2DM have been found to have more adverse clinical outcomes compared to non-diabetic (NDM) patients after Percutaneous Coronary Intervention (PCI). Moreover, prognosis after coronary angioplasty is even worse in insulin-treated T2DM (ITDM) patients when compared to non-insulin treated T2DM (NITDM) patients [2, 3].

Many studies have shown that revascularization performed using Coronary Artery Bypass Surgery (CABG) appears to be a better option compared to PCI in patients with T2DM, particularly in conditions such as multi-vessel CAD, chronic total occlusion and so on. For example, earlier reports based on data from the CARDia (Coronary Artery Revascularization in Diabetes) trial [4] and the 5-year results of the SYNTAX trial [5] indicated significantly higher rates of Major Adverse Cardiovascular and Cerebrovascular Events (MACCEs) associated with PCI compared to CABG in patients with T2DM.

However, even if PCI is expected to be associated with worse clinical outcomes in patients with ITDM, data regarding the long-term adverse clinical outcomes in similar patients revascularized by either CABG or PCI are still controversial. To further support this point, results from the FREEDOM trial showed no significant difference in the magnitude of CABG versus PCI treatment effect in patients with ITDM [2].

Therefore, to solve this issue, we aim to compare the long-term adverse clinical outcomes in patients with ITDM revascularized by either CABG or PCI.

## Methods

### Data sources and search strategy

Medline and EMBASE were searched for Randomized Controlled Trials (RCTs) comparing CABG with PCI in patients with both ITDM and NITDM by typing the words ‘diabetes mellitus, coronary artery bypass surgery and percutaneous coronary intervention’. To further enhance this search, the abbreviations ‘DM, CABG and PCI’ have also been used. References have also been checked for relevant RCTs. No language restriction was applied.

### Inclusion and exclusion criteria

Studies were included if:

- They were RCTs.
- They compared patients with ITDM and NITDM revascularized by either CABG or PCI.
- They reported long-term (≥1 year) adverse clinical outcomes observed in those patients with ITDM.

Studies were excluded if:

- They were not RCTs (excluded if they were observational studies, case studies or meta-analyses).
- Data for patients with ITDM could not be retrieved from the studies.
- They did not compare CABG with PCI in patients with ITDM.
- They had a short-term (<1 year) follow up period.

### Types of participants

All the patients suffered from T2DM and were treated with insulin therapy. The patients were either randomized to undergo revascularization by CABG or PCI.

### Outcomes and definitions

- Adverse clinical outcomes such as all-cause mortality, stroke, MACCEs, Myocardial infarction (MI) and repeated or further revascularization during a long-term follow-up period (≥1 year) were considered as the clinical endpoints in this study. Reported clinical outcomes and follow up periods have been represented in Table 1.

**Table 1:** Reported outcomes and follow up periods

Studies	Follow-up (years)	Clinical outcomes
Banning [8]	1	Death, MI, stent thrombosis, repeated revascularization, MACCEs
Bari [9]	5	Death
Dangas [2]	1, 5	Death, stroke, MI, repeated revascularization, MACCEs
Detre [10]	5	Death
Farkouh [11]	1, 2, 5	Death, MACCEs, MI, stroke, repeated revascularization
Kamalesh [12]	1, 2	Death, stroke, MI, repeated revascularization
Kappetein [13]	5	Death, stroke, MI, repeated revascularization, stent thrombosis
Kapur [4]	1	Death, non-fatal MI, stroke, further revascularization, MACCEs
Lima [14]	10	Death
Soares [15]	1, 2–5	Death

*MI* myocardial infarction, *MACCEs* major adverse cerebrovascular and cardiovascular events
- MACCEs included all-cause death, cerebrovascular accident (CVA), MI or repeat revascularization (subsequent to PCI or CABG).
- Stroke was defined as focal neurological deficits of central origin lasting >72 h, resulting in permanent brain damage or body impairment.
- Death was defined as all-cause death. If data for all cause death was not available, data for cardiac death have been used.

### Data extraction and quality assessment

Data were reviewed and assessed for eligibility and methodological quality by two authors (PKB and ZW). Information regarding study and patients with ITDM, intervention strategies, and the pre-specified clinical outcomes reported and the corresponding follow-up periods was systematically extracted. Disagreements were discussed between the authors, and if the authors could not reach a consensus, disagreements were resolved by the third author (M.H.C).

The bias risk of trials was assessed with the components recommended by the Cochrane Collaboration, including the following criteria: sequence generation of the allocation, allocation concealment, blinding of participants, personnel, and outcome assessors, incomplete outcome data, selective outcome reporting, and other sources of bias [6]. Trials have been carefully assessed and a score ranging from 0 to 12 points has been allocated to specific trials depending on whether they satisfied all the components recommended by the Cochrane Collaboration. Low risk of bias corresponded to a score of 2 in each of these 6 components whereas a score of 0 was given if this evaluation showed a high risk of bias in these RCTs. A score of 1 was reserved for unclear bias. Therefore, if a trial showed ‘low risk bias’ in all the 6 components recommended by the Cochrane Collaboration, a total score of 2 × 6 = 12 would be allocated to it.

### Statistical analysis

The assessment of heterogeneity across the studies was performed using the (a) Cochrane Q-statistic whereby a ‘p value’ less than 0.05 was considered statistically significant and, (b) Cochrane I^2^-statistic which represented the percentage of the total variation across studies that is due to heterogeneity rather than chance whereby an I^2^ value of 0 % indicated no heterogeneity, and an increased heterogeneity was indicated by a larger value. If I^2^ was <50 %, fixed effect was used. However, if I^2^ was >50 %, a random effect has been used. Funnel plots were assessed for publication bias. We calculated odd ratios (OR) and 95 % confidence intervals (CIs) for categorical variables. The pooled analyses were performed with RevMan 5.3 software.

#### Ethics

Ethical approval was not necessary as this study is a Systematic Review and Meta-Analysis.

## Results

### Study selection and general features of the included studies

Study selection, data collection, analysis, and reporting of the results were performed using the recommendations of the PRISMA (Preferred Reporting Items for Systematic Reviews and Meta-Analyses) statement [7].

**Fig. 1:**
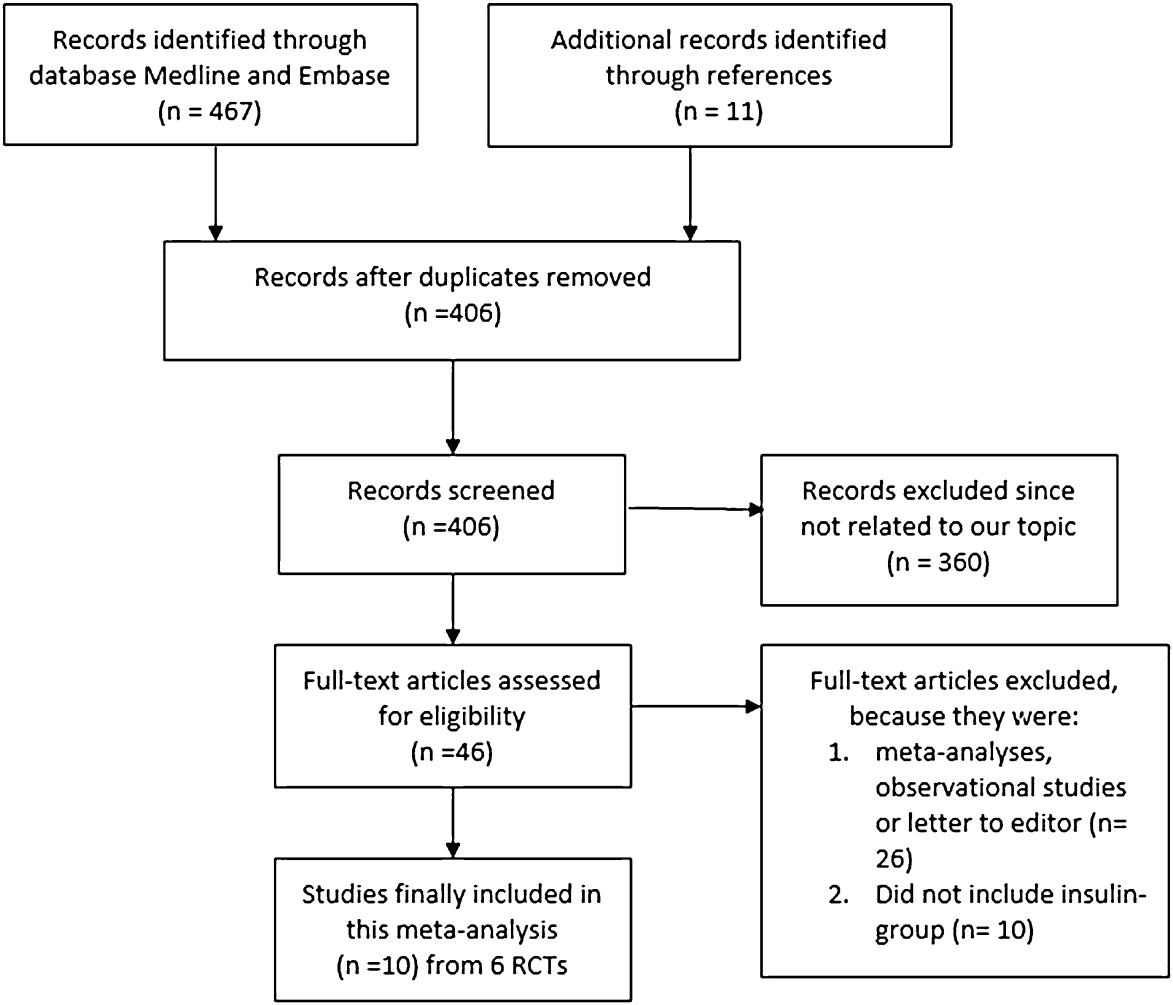
Flow diagram of the study selection. 456 articles were identified from Medline and EMBASE and further 11 relevant articles were identified through reference lists of highly selective studies. After filtering the duplicates, 360 articles were excluded since they were not related to our topic. 46 full-text articles were assessed for eligibility. Meta-analyses, observational studies, and letters to editor were further eliminated (n = 26). Studies including data for patients with ITDM which were unable to be retrieved were also eliminated (n = 10). Finally 6 RCTs involving 10 studies were selected for this systematic review and meta-analysis

Six Trials (involving 10 studies) have been included in this meta-analysis (Table 2). During the selection process, 20 studies comparing CABG with PCI in patients with T2DM were found. However, because data for patients with ITDM could not be retrieved from 10 studies, these studies had been excluded from this analysis. The flow diagram for the study selection has been represented in Fig. 1.

**Table 2:** General features of the included studies

Studies	Trial name	Study type	Region	Randomization period (year)	Patients in CABG group (n)	Patients in PCI group (n)
Banning (2010)	SYNTAX	RCT	England	–	88	88
Bari (1997)	BARI	RCT	Pittsburgh	1988–1991	47	45
Dangas (2014)	FREEDOM	RCT	New York	2005–2010	277	325
Detre (1999)	BARI	RCT	Pittsburgh	1988–1991	80	78
Farkouh (2012)	FREEDOM	RCT	New York	2005–2010	293	322
Kamalesh (2013)	–	RCT	Indiana	2006–2010	45	48
Kappetein (2013)	SYNTAX	RCT	Netherlands	–	93	89
Kapur (2010)	CARDIa	RCT	England	–	99	88
Lima (2013)	MASS II	RCT	Brazil	1995–2000	29	30
Soares (2006)	MASS II	RCT	Brazil	1995–2000	23	19

*CABG* coronary artery bypass surgery, *PCI* percutaneous coronary intervention, *RCT* randomized controlled trials

SYNTAX Trial, BARI Trial, FREEDOM Trial, CARDIa and MASS II Trials were included in this meta-analysis.

These 10 studies [2, 4, 8–15] reported long-term adverse clinical outcomes as their endpoints.

Patient enrollment occurred from the year 1988 to 2010. Randomization of the patients was performed in different medical centers mostly from New York, England and Brazil.

A total number of 1,297 patients with ITDM consisting of 639 patients from the CABG group and 658 patients from the PCI group were included in this meta-analysis. All patients provided signed consents. General features of these included trials have been listed in Table 2.

Six studies reported a follow-up period of 1 year, two studies reported a follow-up period of 2 years, five studies reported a follow-up period of 5 years, one study reported a follow-up between 2 and 5 years and another study with a follow up period of 10 years (Table 1). To avoid repetition, trials were considered.

### Baseline characteristics

Table 3 shows the baseline characteristics of the included studies. A mean age of about 60 years was observed in patients from both groups. With the exception of two studies, similar percentages of males were reported in the CABG and PCI groups. The percentages of hypertensive patients and smokers were also similar in both groups. Overall, there were no significant differences in the baseline features between these patients with ITDM classified in the CABG or PCI group.

**Table 3:** Baseline characteristics of the included studies

Trials	Age (years)	Males (%)	HT (%)	Cs (%)	Cochrane
	CABG/PCI	CABG/PCI	CABG/PCI	CABG/PCI	Bias score
Banning [8]	65.4/65.4	71.0/71.0	69.9/69.9	15.8/15.8	8
Bari (1997)	62.5/62.1	58.0/56.0	66.0/65.0	–	9
Dangas [2]	62.6/62.6	61.3/6.3	87.5/87.5	17.9/17.9	8
Detre [10]	62.3/62.3	57.0/57.0	65.0/65.0	65.0/65.0	9
Farkouh [11]	63.1/63.2	69.5/73.2	–	16.6/14.8	8
Kamalesh [12]	62.1/62.7	99.0/99.0	95.7/96.0	20.6/27.7	8
Kappetein [13]	65.4/65.4	71.0/71.0	70.0/70.0	16.0/16.0	8
Kapur [4]	63.6/64.3	77.9/70.7	76.6/76.6	24.6/24.6	10
Lima [14]	59.0/61.0	72.0/56.0	71.0/72.0	34.0/17.0	8
Soares [15]	60.0/61.0	67.0/54.0	73.0/73.0	–	8

*HT* hypertension, *Cs* current smoker

The bias risk scores were as follow: Seven studies were allocated a score of 8, two studies were allocated a score of 9 and one study was allocated a score of 10. These scores have been listed in Table 3.

### Results of this meta-analysis

**Fig. 2:**
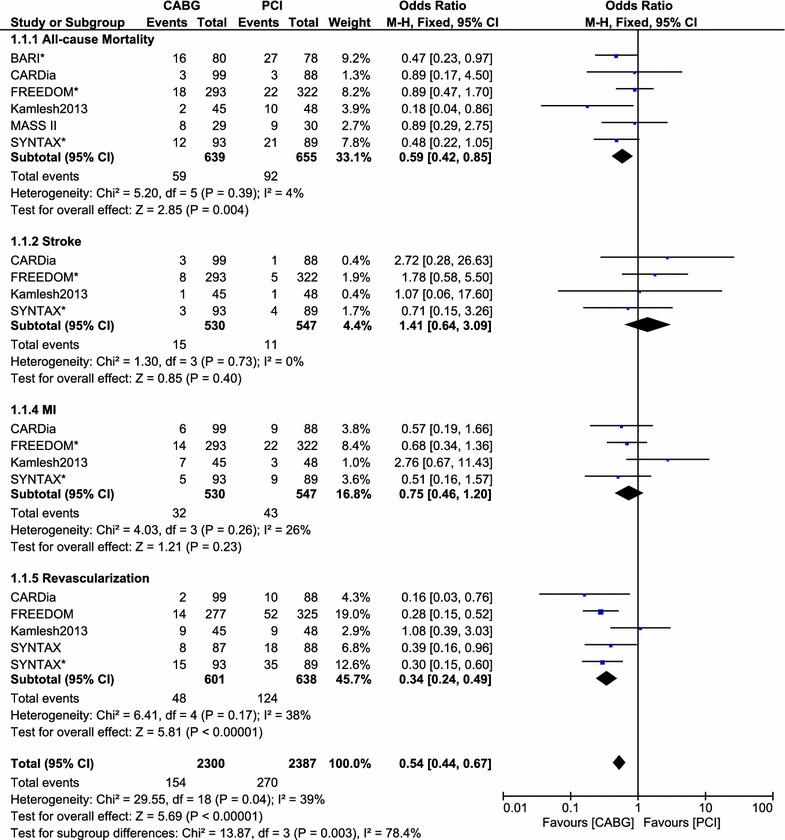
Forest plot comparing the adverse clinical outcomes between the CABG and PCI groups

**Fig. 3:**
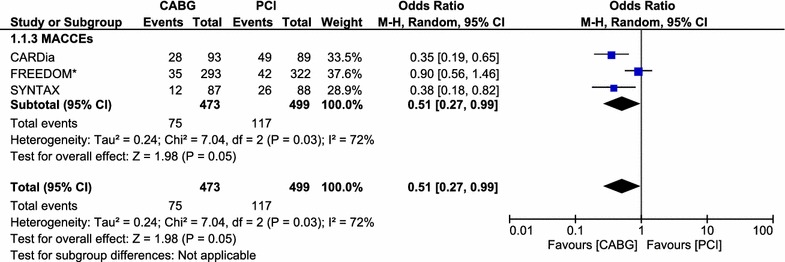
Forest plot comparing major adverse cardiovascular and cerebrovascular events between the CABG and PCI groups

The pooled analysis of these 1207 patients with ITDM showed CABG to be associated with a significantly lower long-term mortality rate with OR: 0.59, 95 % CI 0.42–0.85; P = 0.004. MACCEs and revascularization were also significantly lower in the CABG group with OR: 0.51, 95 % CI 0.27–0.99; P = 0.03 and OR: 0.34, 95 % CI 0.24–0.49; P < 0.00001 respectively. MI insignificantly favored CABG with OR: 0.75, 95 % CI 0.46–1.20; P = 0.23. The rate of stroke was higher in the CABG group with OR: 1.41, 95 % CI 0.64–3.09; P = 0.40. However, the result was not statistically significant. Detailed result of this meta-analysis has been tabulated (Table 4). Results for the adverse clinical outcomes between CABG and PCI, using a fixed effect model (I^2^ < 50 %) have been illustrated in Fig. 2. The result for MACCEs using a random effect model (I^2^ > 50 %) has been illustrated in Fig. 3.

**Table 4:** Result of this meta-analysis

Long-term outcomes	Trials analyzed	OR, 95 % CI	P value	I^2^ %
Mortality	6	0.59 [0.42, 0.85]	0.004	4
MI	4	0.75 [0.46, 1.20]	0.23	26
MACCEs	3	0.51 [0.27, 0.99]	0.03	72
Stroke	4	1.41 [0.64, 3.09]	0.40	0
Revascularization	5	0.34 [0.24, 0.49]	<0.00001	38
Mortality at 1 year	5	1.07 [0.52, 2.19]	0.86	0
Mortality during 5 years	4	0.56 [0.40, 0.79]	0.001	0

*MI* myocardial infarction, *MACCEs* major adverse cerebrovascular and cardiovascular events, *OR* odd ratio, *CI* confidence interval

**Fig. 4:**
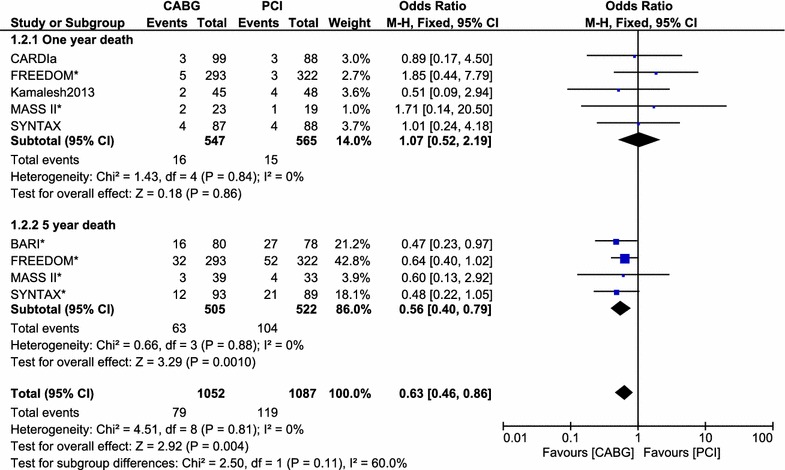
Forest plot comparing mortality at 1 and 5 years between the CABG and PCI groups

At 1 year, mortality rate was similar in both the CABG and the PCI groups. However, during a follow up period of 5 years, mortality was significantly higher in the PCI group with OR: 0.56, 95 % CI 0.40–0.79; P = 0.001. This result has been illustrated in Fig. 4.

**Fig. 5:**
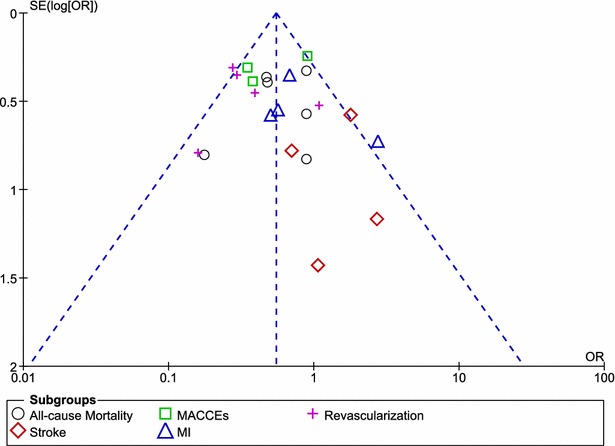
Funnel plot assessing publication bias

For all of the above analyses, sensitivity analysis yielded consistent results. Based on a visual inspection of the funnel plot, there has been no evidence of publication bias for the included studies that assessed all clinical endpoints. The funnel plot has been represented in Fig. 5.

## Discussion

### Aim of this study

Several studies have shown insulin therapy to be associated with worse cardiovascular outcomes in patients with T2DM after revascularization with PCI [3]. However, data regarding the long-term adverse clinical outcomes in patients with ITDM revascularized by either CABG or PCI are still controversial and therefore, we aim to solve this issue in this meta-analysis.

### Results of this meta-analysis

Our results showed long-term mortality rate (9.23 % versus 14.0 %), MACCEs (15.9 versus 23.4 %) and repeated revascularization (7.99 versus 19.4 %) to be significantly lower in the CABG group compared to the PCI group. Mortality during a follow-up period of 5 years was also significantly lower in the CABG group (12.5 versus 19.9 %). However, stroke which was higher in the CABG group with a percentage of 2.83 % compared to 2.01 % in those patients from the PCI group, was not statistically significant in this analysis.

### Other researches supporting our study

Several studies showed similar results with this current meta-analysis. The systematic review and Bayesian network meta-analysis comparing the long-term outcomes between the revascularization techniques of PCI and CABG in patients with T2DM also showed an increased association of cardiovascular outcomes in the PCI group and therefore, concluded that CABG seemed to be the preferred revascularization strategy in such patients especially if long-term survival was to be considered [16]. Another recent meta-analysis of several randomized controlled trials comparing CABG with PCI found significantly lower mortality rates among patients with T2DM revascularized by CABG compared to those patients revascularized by PCI [17]. Moreover, the meta-analysis by Smit et al. comparing CABG with PCI in patients with CAD showed that lower rates of death and repeated revascularization were associated with CABG especially in those patients with T2DM, but however, CABG was associated with a significantly higher risk of stroke in his study [18]. These meta-analyses recently discussed above, compared revascularization by CABG and PCI in patients with CAD or T2DM (consisting of patients with both ITDM and NITDM). Our study focused only on patients with ITDM.

### Is insulin therapy responsible for these adverse cardiovascular outcomes?

Insulin therapy could be one of the reasons responsible for the increased adverse cardiovascular outcomes among patients who underwent revascularization procedures (including both CABG and PCI). Even if the study by Marso et al. showed no significant differences in treatment effect between CABG and PCI in patients with T2DM, the author concluded that insulin therapy remained an independent risk of adverse outcomes in patients with T2DM [19]. Moreover, the prospective registry data of consecutive CABG patients reported mortality to be significantly higher in patients with T2DM treated with insulin therapy, compared to those patients not treated with insulin, or NDM patients [20].

However, insulin therapy is expected to be only partly responsible for these adverse clinical events after revascularization. The review published by Lee et al. which showed a significantly lower rate of mortality and MACCEs among patients with T2DM in the CABG group, concluded that insulin therapy did not affect the clinical outcomes reported in these two revascularization procedures [21]. Also, the study by Liu et al. showed an increased level of HBA1c to be an independent predictor of MACCEs in similar patients [22]. Furthermore, the study by Lopez de Andres et al. showed a higher comorbidity and the female gender to be associated with a high rate of in-hospital mortality in patients with T2DM and NDM [23]. Patients with T2DM often exhibit increased platelet reactivity despite combined treatment with clopidogrel and aspirin after PCI [24]. Patients with ITDM requiring prasugrel after PCI also have higher platelet reactivity compared to patients with NITDM or those without T2DM. This could also contribute to a higher risk of cardiovascular outcomes after revascularization procedures. However, our study was different since it compared CABG and PCI in patients with ITDM.

### Other researches with different results from our meta-analysis

Other studies showed different results compared to ours. For example, the study by Gargiolo et al. comparing the 5 years clinical outcomes between CABG and PCI showed no statistical difference in mortality, MI and stroke between CABG and PCI but showed repeated revascularization which was significantly increased in the PCI group [25]. However, his study only included patients with left main coronary artery disease. Another study, by Hallberg et al. which assessed the association of DM with 16 years survival after revascularization by CABG showed patients without DM to have a similar survival rate with the other patients, while the mortality rate in patients with T2DM started to increase a few years post-CABG [26]. Apart from showing that DM was associated with an increased risk of mortality after CABG, his study also showed an even higher cardiovascular cause of mortality in patients with ITDM compared to those without insulin therapy but however, the study did not compare the adverse cardiovascular outcomes between CABG and PCI. Moreover, Naito et al. compared the mortality rate between CABG and PCI in elderly patients with T2DM complicated with multi-vessel coronary disease and showed no significant difference in the mortality rate between CABG and PCI [27]. But the author stated that the CABG group had more patients with complex coronary lesions which could be responsible for such an outcome.

Even if the rate of stroke was not statistically significant in our study, a few other RCTs have reported a significant increased risk of stroke in those patients revascularized by CABG compared to PCI. However, all these studies were not powerful enough to examine strict differences in the risk of stroke. Two prior meta-analyses have analyzed the risk of stroke post-CABG or post-PCI, with conflicting results [28, 29]. But, another meta-analysis including 19 trials with 10,944 patients randomized to CABG and PCI showed a real association of an increased risk of stroke at 30 days and at mid-term follow up in the CABG group compared to the PCI group [30]. Post-CABG versus post-PCI stroke rates (post-CABG stroke rate at 1 year was 1.83 %; with OR 1.67). Nevertheless, the increased stroke rate following CABG compared to PCI was independent of ITDM status (reported as 5-year rates of 7.5 vs. 3.7 % for CABG and PCI in patients with ITDM; and 4.3 vs. 1.7 % for CABG and PCI in patients with NITDM) and could mean an association of stroke with CABG and not with the insulin therapy.

### Novelty in this study

This study has strictly been conducted in patients with ITDM. Several studies have compared CABG with PCI in patients with CAD or T2DM (including a combination of patients with both ITDM and NITDM). However, since patients with ITDM are more complicated patients with several co-morbidities, we have conducted a meta-analysis on this particular population of patients with T2DM.

Finally, similar to the FREEDOM trial which clearly showed revascularization by CABG to be superior compared to PCI in patients with T2DM, our study which also showed CABG to be better than PCI in patients with ITDM, also suggests a comparative analysis of the new incoming stents which warrant further research [31].

Our study is expected to satisfy all the requirements for a meta-analysis, in terms of low heterogeneity in almost all of the different subgroups, absent publication bias, and sensitivity analysis, and provides robust scientific validity to our findings, which can assist informed decision making by patients and physicians when deciding on the optimal strategy for revascularization in CAD patients with ITDM.

### Limitation

Due to the small population size, the result of this study could be restricted to an extent. Moreover, these RCTs included patients with ITDM with different clinical conditions or complications. For example, the CARDia and FREEDOM trials included patients with symptomatic multi-vessel CAD and multi-vessel CAD with or without symptoms respectively whereas the SYNTAX trial included patients with left main coronary disease and/or three vessel disease. The BARI trial included patients with severe CAD. Variable degree of complications in these patients could have an effect on our results. However, despite these limitations, our data point to the urgent need for comprehensive comparison between these two revascularization strategies.

## Conclusion

Compared to PCI, CABG was associated with a significantly lower mortality rate, MACCEs and repeated revascularization during this long-term follow up period in patients with ITDM. However, even if a higher rate of stroke in the CABG group was not statistically significant in our result, new researches with larger number of randomized patients are required to completely solve this issue.

## Abbreviations

CABG: coronary artery bypass grafting
PCI: percutaneous coronary intervention
T2DM: type 2 diabetes mellitus
ITDM: insulin-treated diabetes mellitus
MACCEs: major adverse cardiovascular and cerebrovascular events
